# Adipose-derived mesenchymal stem cells and their derivatives in inflammatory skin diseases: a systematic review

**DOI:** 10.3389/fimmu.2025.1617157

**Published:** 2025-08-22

**Authors:** Mateusz Matwiejuk, Agnieszka Mikłosz, Hanna Myśliwiec, Adrian Chabowski, Iwona Flisiak

**Affiliations:** ^1^ Department of Dermatology and Venereology, Medical University of Bialystok, Bialystok, Poland; ^2^ Department of Physiology, Medical University of Bialystok, Bialystok, Poland

**Keywords:** ADMSCs, small extracellular vesicles, exosomes, skin diseases, psoriasis, atopic dermatitis, localized scleroderma, acne vulgaris

## Abstract

Adipose-derived mesenchymal stem cells (ADMSCs) offer a multifaceted approach to treating immune-mediated skin diseases by modulating the immune system and promoting tissue regeneration. Specifically, their ability to differentiate into multiple cell types such as keratinocytes and fibroblasts, modulate immune responses, and release growth factors and cytokines underscores their potential in treating a wide range of immune-related skin conditions. ADMSCs significantly reduced various aspects of psoriasis, including scaling, thickness, and erythema. Moreover, cell-free therapy has even better therapeutic potential. It has been shown that ADMSC-derived exosomes can effectively alleviate pathological symptoms of atopic dermatitis, including clinical score, serum IgE levels, eosinophil amount, and infiltration of immune cells in skin lesions. This systematic review summarizes the most relevant preclinical and clinical studies on the therapeutic use of ADMSCs and their small extracellular vesicles in the treatment of common skin diseases like psoriasis, atopic dermatitis, localized scleroderma and acne vulgaris.

## 1 Introduction

Psoriasis and atopic dermatitis (AD) are two common immune-mediated inflammatory skin diseases that have a significant impact on the patient’s quality of life. They can be associated with several comorbidities, such as depressive illness, psoriatic arthritis or cardiovascular disease (1). Although conditions are widespread worldwide and occur in adults with a prevalence ranging from 0.27% to 11.4% for psoriasis and from 2.1% to 4.9% for AD, atopic dermatitis is more common in children than in adults (2). Recently, there has been progress in understanding the mechanisms underlying these multifactorial diseases, which has led to the development of various treatment options. Diseases can be treated with immunosuppressive drugs, but with long-term treatment, a number of side effects and drug resistance have been observed. Therefore, the need for new, more effective therapies for these chronic inflammatory skin diseases is crucial. Mesenchymal stem cells (MSCs) and their derivatives are widely used in treatment of the immune system diseases, with few side effects, making them a promising treatment for chronic inflammatory skin diseases (3). Early research suggests stem cells may be a safe and effective treatment for psoriasis and AD, but it’s not yet an approved treatment as more research is needed. MSCs represent attractive cell therapy agents for the treatment of inflammatory skin diseases due to their regenerative capacity, paracrine activity, and immunomodulatory and immunosuppressive properties. MSCs are multipotent stem cells characterized by their capacity to differentiate into various cell types including adipocytes, osteocytes, hepatocytes, chondrocytes, and neurons (4). Moreover, MSCs exert therapeutic effects in virtue of their abilities to secrete a variety of signaling molecules, including cytokines (e.g. interleukins - IL-6, IL-8, INF- γ), growth factors (e.g. hepatocyte growth factor (HGF), vascular endothelial growth factor (VEGF), insulin-like growth factor-1 (IGF-1), granulocyte colony‐stimulating factor (G‐CSF), granulocyte macrophage colony‐stimulating factor (GM‐CSF), nerve growth factor (NGF), or insulin‐like growth factor 1 (IGF‐1)) and other mediators (5). MSCs can originate from different tissues, including adipose tissue, bone marrow, liver, blood, skeletal muscle, skin, placenta or umbilical cord. Nevertheless, MSCs from different origin have distinct characteristics, impacting their suitability for specific clinical applications, including psoriasis and atopic dermatitis treatment. Although both bone marrow-derived MSCs (BM-MSCs) and adipose tissue-derived MSCs (ADMSCs) have become the most commonly used stem cells in cell therapy, ADMSCs are increasingly favored. ADMSCs exhibit typical features of MSCs such as remarkable self-renewal capacity, paracrine activity and the ability to differentiate into different cell types including mesodermal lineages (adipocytes, osteoblasts, chondrocytes, fibroblasts, and myocytes) as well as non-mesodermal lineages (neurons, hepatocytes, endothelial cells, and cardiomyocytes) (7). This makes ADMSCs a promising source for treatment various diseases like non-alcoholic fatty liver disease, liver cirrhosis, ischemic stroke, multiple sclerosis, Crohn’s disease (8–11). Furthermore, ADMSCs play a pivotal role in wound repair, where by enhancing cell proliferation they can stimulate cell growth and division, which is crucial for tissue regeneration and repair (12). For instance, ADMSCs show promise in treating acne vulgaris due to their ability to reduce inflammation and potentially promote skin regeneration. They can modulate the inflammatory response associated with acne, including reducing the formation of neutrophil extracellular traps (NETs) and inhibiting the NLRP3 inflammasome pathway, both of which are involved in acne development. Additionally, ADMSCs can help improve skin quality by increasing collagen production and potentially reducing the appearance of acne scars (13). Moreover, ADMSCs can suppress the overactive immune response that drives scleroderma, helping to reduce inflammation and tissue damage. They can inhibit the excessive production of collagen and other substances that lead to fibrosis, the hallmark of scleroderma (14).

**Figure 1:**
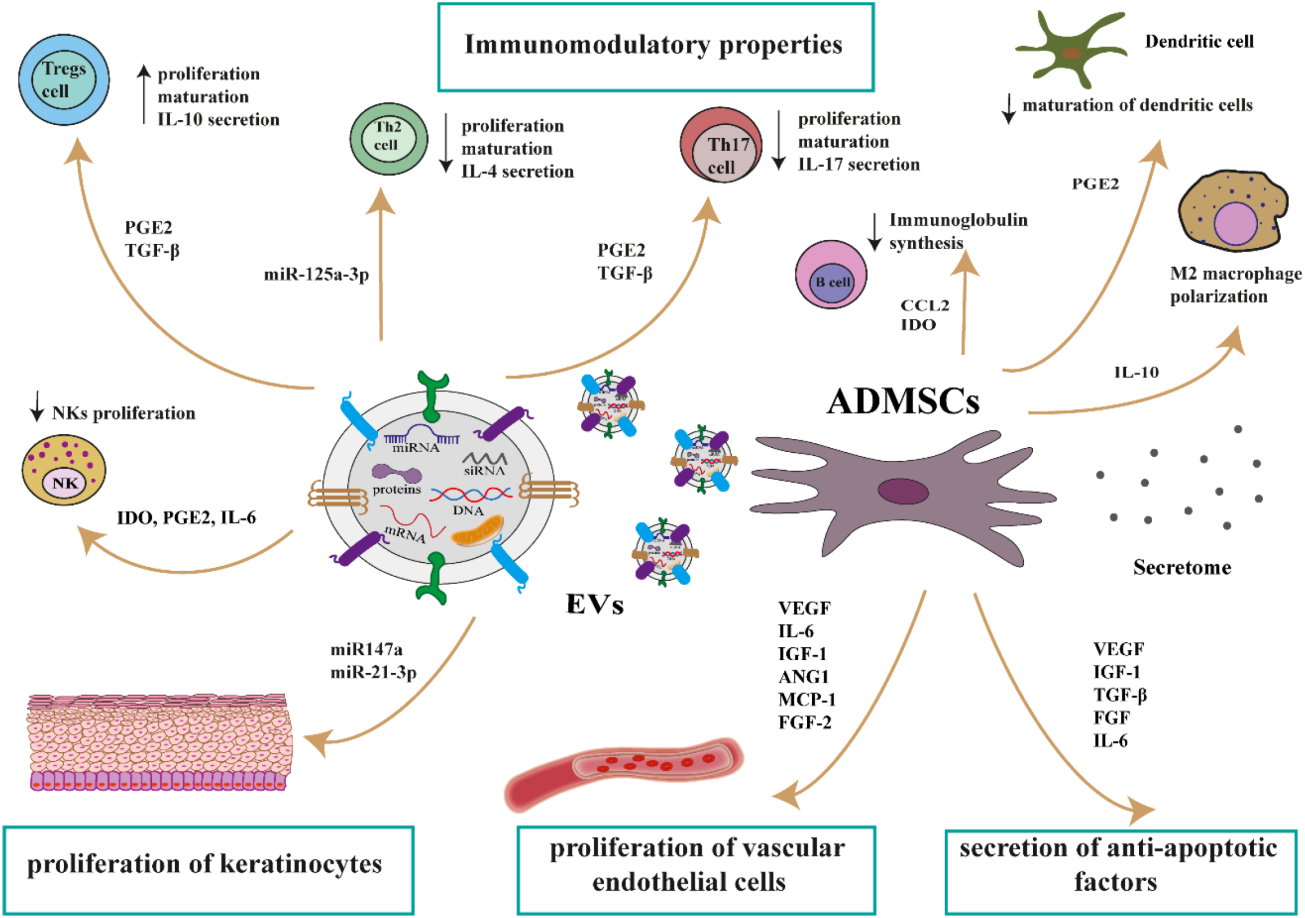
The potential mechanism of ADMSCs action in the treatment of inflammatory skin diseases (e.g., atopic dermatitis, psoriasis). ADMSCs, adipose-derived mesenchymal stem cells; ANG1, angiopoietin-1; CCL2, chemokine (C-C motif) ligand 2; CCL3, chemokine (C-C motif) ligand 3; CCL4, chemokine (C-C motif) ligand 4; CCL5, chemokine (C-C motif) ligand 5; CCL6, chemokine (C-C motif) ligand 6; CX3CL1, chemokine (C-X3-C motif) ligand 1; CXCL5, chemokine (C-X-C motif) ligand 5; FGF, fibroblast growth factor; IDO, indoleamine 2,3-dioxygenase; IGF-1, insulin-like growth factor 1; IL-10, interleukin 10; IL-6, interleukin 6; MCP-1, monocyte chemoattractant protein 1; NK, natural killer cells; PGE2, prostaglandin 2; TGF-β, tumor growth factor β; VEGF, vascular endothelial growth factor.

ADMSCs-based therapies are promising for treating chronic inflammatory skin diseases due to their immunomodulatory and anti-inflammatory properties. They can modulate the immune system by interacting with various immune cells and secreting factors that reduce inflammation. This makes them a potential therapeutic option for conditions like atopic dermatitis and psoriasis, where inflammation plays a key role. ADMSCs exert their therapeutic effects on inflammatory skin diseases (e.g., atopic dermatitis, psoriasis) through cellular interactions and paracrine effects (**Figure 1**). ADMSCs exhibit potent immunomodulatory properties, influencing both the innate and adaptive immune systems through the release of EVs and soluble cytokines. They can modulate the immune response by promoting M2 macrophage polarization and T regulatory cell (Treg) proliferation (via IL-10), inhibiting the proliferation of CD8+ and CD4+ T lymphocytes and natural killer (NK) cells (via IDO, PGE2, TGF-β, IL6), inhibiting dendritic cells (DCs) differentiation (via PGE2) and suppressing B cell development (via CCL2, IDO). In addition, ADMSCs secrete a variety of chemoattractant factors (e.g., CCL2, CCL3, CCL4, CCL5, CCL6, CX3CL1, CXCL5), anti-apoptotic factors (e.g., VEGF, IGF-1, FGF, TGF-β, IL-6), and angiogenesis promoting factors (e.g., VEGF, IGF-1, ANG-1, MCP-1) (15). Moreover, EVs miR-125a-3p inhibits the proliferation and activation of Th2 cells, whereas miR-147a and miR-21-3p promote the proliferation of keratinocytes and vascular endothelial cells, ultimately facilitating the healing of skin lesions (16). Finally, ADMSCs and EVs secrete a broad range of anti-apoptotic factors such as VEGF, IGF-1, TGF-β, FGF.

ADMSCs offer some advantages such as easier collection, greater availability, and superior immunomodulatory properties compared to other MSCs. Patients can be treated using autologous or allogeneic ADMSCs, which is associated with a low risk of cellular rejection and eliminates ethical and legal issues. Furthermore, ADMSCs tend to proliferate at a higher rate as compared to BM-MSCs, allowing for faster expansion in culture (17). However, the rate of proliferation is highly dependent on the adipose tissue deposit, as MSCs derived from subcutaneous adipose tissue have a faster proliferation rate than those originating from the omental region. Recent studies suggested that ADMSCs exhibit greater resilience to harsh conditions, such as oxidative stress and hypoxia, compared to BM-MSCs. Specifically, ADMSCs demonstrate enhanced survival rates and angiogenic potential under these conditions (18). These advantages, combined with minimal immunogenicity and high immunoregulatory capacity, make them attractive for clinical applications. On the other hand, umbilical cord MSCs (UC-MSCs) also exhibit some advantages such as non-invasive harvesting, high proliferative capacity with low immunogenicity, which makes them a good candidate for treating immune diseases (19). However, the main disadvantage of UC-MSCs is their limited availability, as they can only be harvested immediately after birth. While ADMSCs offer promising therapeutic potential, they are not without their drawbacks. These include potential adverse events, limitations in their therapeutic efficacy, and challenges related to their use in specific patient populations. For instance, hyperglycemic environment can hinder ADMSCs’ ability to differentiate and proliferate, ultimately affecting their overall functionality (20). Additionally, differences in the efficacy of ADMSCs may be influenced by donor characteristics like genetics or lifestyle. A significant hurdle in stem cell therapy is the poor survival and engraftment of transplanted cells. To address this, researchers are actively developing strategies to enhance cell survival and retention within the recipient’s body.

In addition to ADMSCs, their derived exosomes have shown potential in the treatment of inflammatory skin diseases. Exosomes are small extracellular vesicles (sEVs), that play a crucial role in cell-to-cell communication, both locally and distantly and facilitate the transfer of biological information between cells (21). Exosomes contain a variety of components, including proteins, mRNAs, miRNAs, and lipids. They can deliver their cargo to target cells through various mechanisms, including ligand/receptor interaction, and membrane fusion (22). There are few advantages of MSC-derived exosomes as a potential therapeutic option compared to stem cells themselves. Firstly, exosomes are less likely to trigger an immune response, reducing the risk of rejection. Secondly, exosomes do not pose a risk of tumor formation. Thirdly, exosomes have a generally favorable safety profile, making them suitable for clinical use. Exosomes are commonly used in wound healing, they support angiogenesis, enhance collagen fiber formation and reduce inflammation (23).

Recent studies indicate that MSCs undergo rapid apoptosis after administration and their therapeutic effects are related to apoptotic vesicles released from apoptotic MSCs (MSC-ApoVs) (24). It has been shown that MSC-ApoVs can effectively control the inflammatory immune response, particularly by inhibiting Th17 cell activity and shifting the cytokine balance towards anti-inflammatory effects. This modulation suggests a potential role of MSC-ApoVs in preventing or alleviating conditions driven by excessive inflammation, such as inflammatory skin diseases (25). ADMSC-ApoVs significantly decrease the release of pro-inflammatory cytokines like interferon-γ (IFN-γ), thymic stromal lymphopoietin (TSLP), and IL-4, which are known to be elevated in atopic dermatitis. In addition, ADMSC-ApoVs led to a reduction in the infiltration of inflammatory dendritic cells in the epidermis. In AD, inflammatory dendritic cells infiltrate the skin and contribute to allergic inflammation. A relief of itching, erythema and skin xerosis was noted (26). Thus, it seems that ADMSC-ApoVs may be a promising new therapeutic approach for the treatment of dermatitis. While initial studies have highlighted their beneficial properties, further studies are crucial to fully understand their mechanisms and establish their efficacy and safety in the clinical setting.

Although preclinical and clinical studies confirm that ADMSCs and their derivatives are effective therapeutic agents in the treatment of skin diseases, the precise mechanisms by which these cells support the treatment of dermatological diseases require further investigation. This systematic review will investigate the mechanisms and recent findings related to ADMSCs and their derivatives affect common immune-related skin diseases such as psoriasis, atopic dermatitis, localized scleroderma, and acne vulgaris. The review also aims to identify knowledge gaps, suggest future research directions, and provide current insights that can help develop targeted therapies and improve patient outcomes.

## 2 Materials and methods

A medical literature search of PubMed (2010–present), conducted in the winter of 2025, was performed using appropriate terms without date limitations. The main object of the research was to identify the therapeutic effect of ADMSCs in common skin diseases. Medical subject headline terms included “Adipose-derived mesenchymal stem cells”, “ADMSC-derived small extracellular vesicles”, “ADMSCs in psoriasis”, “ADMSCs in atopic dermatitis”, “ADMSCs in acne vulgaris”, “ADMSCs in localized scleroderma”, ÁDMSCs in systemic sclerosis”, “ADMSC-derived small extracellular vesicles in psoriasis”, “ADMSC-derived small extracellular vesicles in atopic dermatitis”, “Exosomes from ADMSCs in psoriasis”, “Exosomes from ADMSCs in atopic dermatitis”, “ADMSC-derived small extracellular vesicles in localized scleroderma”, “ADMSC-derived small extracellular vesicles in systemic sclerosis”, “ADMSC-derived small extracellular vesicles in acne vulgaris”. Data were collected on the characteristics of the studies (authors, year of publication), the population (sample size, mean age and sex), description of the intervention, key observation.

**Figure 2:**
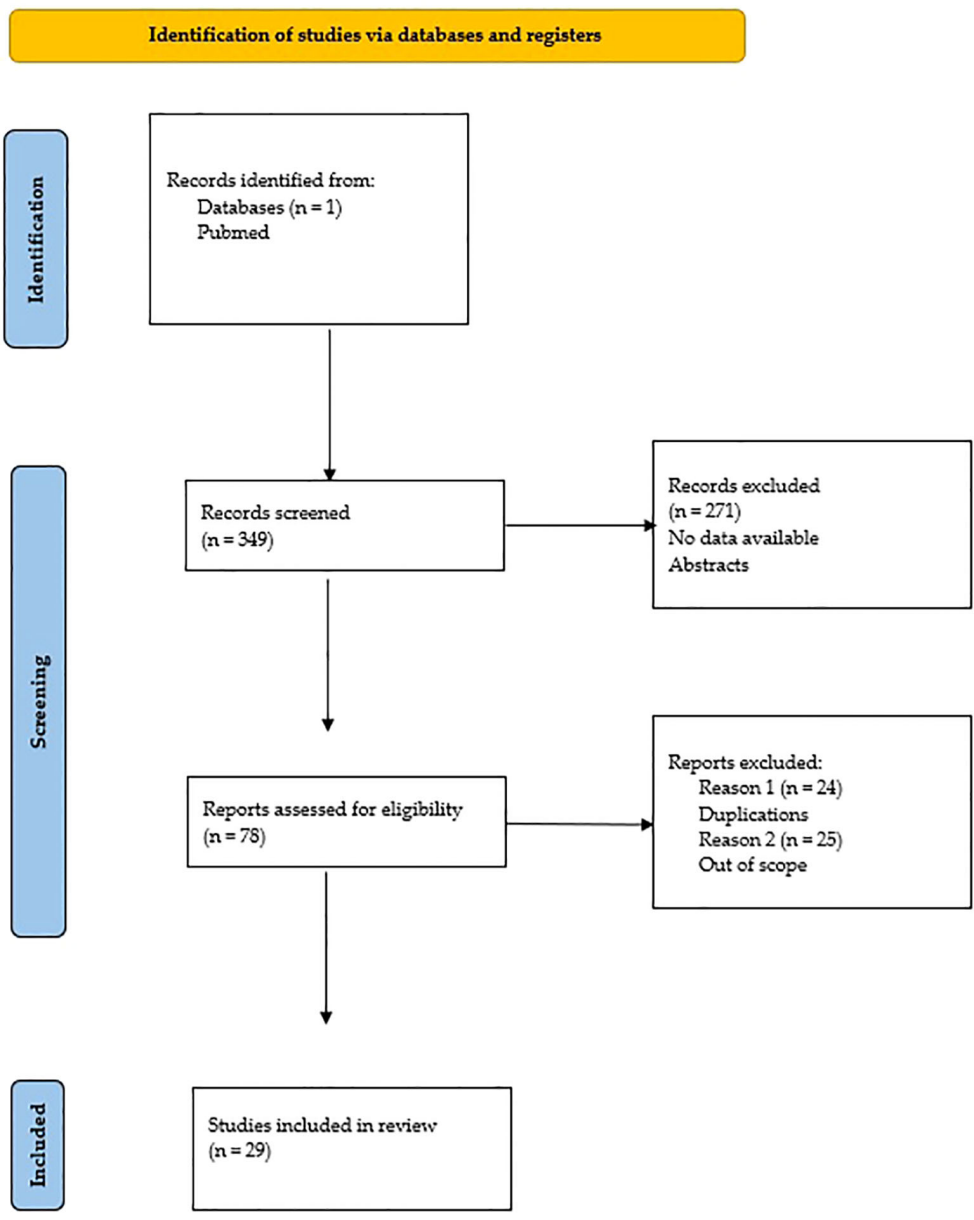
The search process.

According to **Figure 2**, the search resulted in the screening of 97 records, of which 47 were assessed for eligibility and 40 were ultimately included in the qualitative synthesis.

Non-English publications, papers with low clinical significance, and duplicated publications were excluded from the analysis. Originally, human and animal studies were included in this systematic review. The results of the search strings were combined, and duplicates were removed. The risk of bias assessment was carried out by two blinded reviewers R1 (M.M) and R2 (A.M.) and disagreements were resolved by the third reviewer R3 (H.M). Any disagreements concerning the inclusion or exclusion of studies were addressed through discussions among the authors until a unanimous decision was achieved. Finally, the selected eligible articles were fully reviewed.

## 3 Results and discussion

### 3.1 Psoriasis

#### 3.1.1 Adipose-derived mesenchymal stem cells

De Jesus et al. (12) explored the use of ADMSCs for psoriasis vulgaris (PV) and psoriatic arthritis (PsA). Firstly, PsA patient previously unresponsive to standard treatments experienced a significant decrease in Psoriasis Area and Severity Index (PASI) score (from 21.6 to 9.0) after two ADMSCs infusions, indicating improved skin condition. Autologous ADMSCs were infused intravenously at a dose of 0.5–3.1 million cells/kg. However, joint pain persisted, requiring additional treatment with etanercept and infliximab. Secondly, a PV patient dependent on methotrexate substantially reduced PASI score (from 24.0 to 8.3) after three ADMSCs infusions. This clinical improvement was maintained for 9.7 months without the need for methotrexate. Although a marginal decrease in serum tumor necrosis factor-α (TNF-α) levels was observed, a significant reduction (3.5- to 5-fold) in reactive oxygen species (ROS) activity was noted. The ROS levels correlated with clinical improvement, suggestting that ADMSCs may exert their therapeutic effects, at least partially, through modulation of oxidative stress. Importantly, no serious adverse events were reported for either patient following MSCs infusions. However, a small sample size, especially in the case of only two participants, is a significant limitation of a case report. A small sample size limits the generalizability of the findings to a larger population. It also increases the risk of random variability affecting the results, potentially leading to inaccurate conclusions. Furthermore, small samples may not be representative of the broader population, making it difficult to draw meaningful inferences (12) (**Table 1**).

**Table 1:** Summary of the studies on ADMSCs and ADMSCs-derived small extracellular vesicles in psoriasis.

Author	Year	Population	Key observation
ADMSCs in psoriasis			
De Jesus et al. (12)	2016	2 patients with psoriasis; Dose of ADMSCs: 0,5-3.1 million cells/kg i.v.	ADMSCs significantly decreased PASI score in psoriatic patients
Comella et al. (27)	2018	1 patients with psoriasis; Dose of SVF: 3 ml i.v.	ADMSCs in SVF improved psoriatic skin lesions
Seetharman et al. (6)	2019	1 patients with psoriasis Dose of ADMSCs: 5×10^6^ i.v.	ADMSCs therapy led to a complete disappearance of severe psoriatic plaques within one month
Bajouri et al. (21)	2023	3 male patients with psoriasis and 2 female patients with psoriasis; Dose of ADMSCs: 1×10^6^ or 3×10^6^ cells/cm^2^ i.v.	ADMSCs therapy significantly reduced various aspects of psoriasis, including scaling, thickness, and erythema
Yao et al. (22)	2021	8 patients with psoriasis; Dose of ADMSCs: 0.5×10^6^ cells/kg i.v.	ADMSCs is promising modality in the treatment of psoriasis
ADMSC-derived small extracellular vesicles in psoriasis			
Kim et al. (23)	2023	HaCaT Dose of ADMSCs: 3.7×109 cells/cm2 i.v.	ADMSCs exosomes have a suppressive effect on psoriasis serum exosome-induced inflammation and oxidative stress
Mohseni Meybodi et al. (28)	2024	12 patients with plaque psoriasis; Dose of ADMSCs: 200 μg	ADMSC-derived exosomes have a dose-dependent anti-inflammatory effect in the treatment of psoriasis

PV, psoriasis vulgaris; PsA, psoriatic arthritis; PASI, Psoriasis Area and Severity Index; ROS, reactive oxygen species; SVF, stromal vascular fraction; PSSI, Psoriasis Scalp Severity Index; TNF-α, tumor necrosis factor – alpha; IFNγ-sEVs, interferon-gamma-enriched extracellular vesicles; HaCaT, *human epidermal keratinocytes*.

Similarly, Comella et al. (27) demonstrated the safety and potential clinical utility of ADMSCs in the treatment of psoriasis. A single transplantation of ADMSCs resulted in the complete resolution of psoriasis plaques within one month. Importantly, a positive effect persisted for at least 12 months, indicating a long-term efficacy of this approach. The PASI score decreased dramatically from 50.4 to 0.3, demonstrating a substantial improvement in psoriasis severity. Following a case study showing the safety and feasibility of stem cell-based therapy, future research should focus on large, double-blind clinical trials to assess long-term effects, establish efficacy, and address any potential safety concerns related to immune system overactivity. A study with only one patient has a major drawback: it cannot be generalized to a larger population (27) (**Table 1**).

The results presented by Seetharman et al. (6) further confirm the potential of ADMSCs in the treatment of psoriasis, especially in severe cases. ADMSCs therapy led to a complete disappearance of severe psoriatic plaques within one month. The Psoriasis Scalp Severity Index (PSSI) score, which specifically assesses scalp psoriasis, decreased dramatically from 28 to zero. The positive effects persisted for at least 6 months, suggesting a long-lasting benefit. Similarly, to the above-mentioned studies, the major limitation of the study is the sample size of 1 participant (6) (**Table 1**).

Recent findings by Bajouri et al. (21) reinforce the emerging role of ADMSCs in psoriasis management. ADMSCs therapy significantly reduced various aspects of psoriasis, including scaling, thickness, and erythema. The PASI score, a widely used measure of psoriasis severity, decreased significantly, indicating a substantial improvement in the overall condition. No major adverse effects were reported, suggesting that ADMSCs are generally safe and well-tolerated. The small sample size of five participants, with two receiving different doses of ADMSCs, is a significant limitation of this research. This limits the generalizability of the findings and makes it difficult to draw firm conclusions about the efficacy and safety of ADMSC treatment (21) (**Table 1**).

Furthermore, Yao et al. (22) in a single-center, open-label pilot study investigated the safety and efficacy of ADMSCs in seven patients with moderate to severe psoriasis. Two out of seven patients achieved a 50% improvement in their PASI score (PASI-50) after one year of follow-up, and one patient maintained PASI-50 for almost three years without any further treatment. Additionally, the procedure was deemed safe, with no severe adverse events related to the use of allogeneic ADMSCs. A limitation of the study is that only four participants completed the trial, and two patients completed the one-year follow-up (22) (**Table 1**).

#### 3.1.2 ADMSC-derived small extracellular vesicles

Kim et al. (23) showed that ADMSCs-derived exosomes have a suppressive effect on psoriasis serum exosome-induced inflammation and oxidative stress. This suppression is achieved by regulating autophagy in keratinocytes. ADMSCs exosomes reduced the production of proinflammatory cytokines (IL-1β, IL-6 and TNF-α) and oxidative stress. Moreover, they restored autophagy in high-sensitivity *of human epidermal keratinocytes* (HaCaT) cells that had been treated with psoriasis serum-derived exosomes. The study’s reliance on *in vitro* experiments with HaCaT cells is a limitation because the findings might not fully represent the complex pathogenesis of psoriasis in a living organism (*in vivo*). Furthermore, six control participants and six psoriasis patients were included for exosome isolation, further limiting the power of the study (23) (**Table 1**).

Mohseni Meybodi et al. (28) showed that autologous ADMSCs-derived exosomes exert a dose-dependent anti-inflammatory effect in the treatment of psoriasis. In patients receiving 200 µg of exosomes in a single dose, a significant reduction in erythema and induration was observed. Moreover, higher doses (100 μg or 200 μg) were associated with greater reductions in pro-inflammatory markers (IL17, CD3, IFNγ, IL23, TNFα) and increased the expression of the anti-inflammatory marker IL10. The treatment appears to modulate immune cell populations, favoring regulatory T cells (FOXP3+) over pro-inflammatory cells (IL17+ and CD3+). However, limitations of this research included small sample size (only 12 participants) and possible poor treatment protocol. These factors can introduce bias and reduce the reliability of the study’s findings. Thus, higher doses or multiple injections with specific intervals can increase confidence (28) (**Table 1**).

### 3.2 Atopic dermatitis

#### 3.2.1 Adipose-derived mesenchymal stem cells

Shin et al. (29) provided compelling evidence that human adipose tissue-derived mesenchymal stem cells (hADMSCs) can effectively alleviate atopic dermatitis by modulating B lymphocytes function. Intravenous administration of hADMSC at both low dose; 2 × 10^5^ as well as at high dose; 2 × 10^6^ led to a decrease in both gross and histological signs of AD. Serum IgE level, a key marker of allergic responses, was also reduced. Furthermore, hADMSCs significantly inhibited the proliferation and maturation of B lymphocytes. The inhibition was mediated through cyclooxygenase (COX)-2 signaling, suggesting a potential anti-inflammatory mechanism. Only animals (mice) were used in the research model, which affects the generalizability of the results to humans. While mice are a common model organism in research due to their genetic similarity and ease of use, they differ from humans in many biological aspects, potentially leading to inaccurate predictions about human outcomes (29) (**Table 2**).

**Table 2:** Summary of the studies on ADMSCs, ADMSCs-derived small extracellular vesicles in atopic dermatitis, systemic sclerosis, localized scleroderma, and acne vulgaris.

Author	Year	Population	Key observation
ADMSCs in atopic dermatitis			
Shin et al. (29)	2017	NC/Nga mice with AD, *Dermatophagoides farinae-induced murine AD; Dose of ADMSCs: low dose:* 2 × 10^5^, high dose: 2 × 10^6^ i.v.	hADMSCs can effectively alleviate atopic dermatitis by modulating B lymphocytes function. ADMSCs-based therapy reduced the gross and histological signatures of AD, for instance, excoriation, edema, dryness, and erythema. Additionally, serum IgE level was also reduced.
Hall et al. (30)	2010	Canine with AD; Dose of ADMSCs: 1.3 million cells/kg i.v.	ADMSCs did not have a significant impact on the clinical signs of canine atopic dermatitis
ADMSC-derived small extracellular vesicles in atopic dermatitis			
Cho et al. (31)	2023	Canine with AD; Dose of EVs: 7.45 × 10^8^ (low dose), 2.98 × 10^9^ (mid-dose), and 1.19 × 10^10^ (high dose) particles/20 g s.c.	cASC-EVs alleviates AD-like dermatitis symptoms like skin barrier defects, inflammation and itchiness. Moreover, it was observed a reduction in serum IgE levels
Kim et al. (32)	2022	Six-week-old male BALB/c mice; Dose of EVs: cASCs (2 × 10^6^ cells/head) or cASC-EVs (2 × 10^10^ particles/head s.c.	cASC-EVs significantly reduced the level of inflammatory cytokines associated with AD (IL-4, IL-13, IL-31, RANTES). In addition, they promoted skin barrier repair by reducing TEWL, increasing stratum corneum hydration, and upregulating the expression of epidermal differentiation proteins ((AKT and BCL-2).
Cho et al. (33)	2018	NC/Nga mice; Dose of exosomes: 0.14 μg/head, 1.4 μg/head, and 10 μg/head i.v./s.c.	ADMSCs exosomes reduced various pathological symptoms of AD, including clinical score, serum IgE levels, eosinophil count, and infiltration of immune cells in skin lesions.
Shin et al. (34)	2020	AD mice; Dose of exosomes: 1, 3, and 10 μg/head s.c./topically	ADMSC-exosomes improved epidermal barrier function in patients dealing with AD
Roh et al. (35)	2024	HaCaT, HDF, HMC-1 cell lines; Dose of exosomes: 25 μg/cm^2^	ADMSC-exosomes alleviated inflammation and skin barrier damage in AD-like triple-cell model
Park et al. (36)	2021	Two patients with AD; Dose of exosomes: 2.0 × 10^9^ particles/mL i.v.	ADMSCs-derived exosomes reduced dupilumab facial redness in patients with severe atopic dermatitis.
ADMSC-derived small extracellular vesicles in systemic sclerosis			
Rozier et al. (37)	2021	Healthy and human SSC fibroblasts; Dose of EVs: 25 to 400 ng	ASC-EVs demonstrated anti-fibrotic properties. ADMSC-derived EVs had anti-fibrotic and pro-remodeling effects on Tβ-Fb. It was spotted a decreased expression of *COL1A1, COL3A1, αSMA*, *IL-6* and the upregulation of *MMP1/TIMP1.*
Kim et al. (38)	2024	DBA/2 mice; Dose of EVs: 1 ng/mL or 2.5 ng/mL ng/ml	ASC-EVs decreased both the thickness of dermal tissue and the number of α-SMA-expressing cells (markers of fibrosis)
ADMSC-derived small extracellular vesicles in localized scleroderma			
Wang et al. (39)	2023	LSF; Dose of exosomes: 1× 10^6^ cells s.c.	ADMSC-exosomes can prevent the bioactivity of LSF via reducing several proteins taking part in process of fibrosis.
ADMSC-derived small extracellular vesicles in acne vulgaris			
Kwon et al. (40)	2020	25 patients with acne; Dose of exosomes: 9.78×10^10^ particles/ml (for the day of FCL treatment) or 1.63×10^10^ particles/ml i.v.	ADMSC-exosome-treated sides showed a greater reduction in acne scar severity. They can stimulate the movement and growth of skin cells, which is essential for wound healing and tissue regeneration. By reducing the levels of inflammatory cytokines, they can help to reduce inflammation and promote healing.

hADMSCs, human adipose tissue-derived mesenchymal stem cells; COX, cyclooxygenase; SSC, systemic sclerosis; CTGF, connective tissue growth factor; cASC-EVs, canine ADMSC-derived extracellular vesicles; CADESI-03, Canine Atopic Dermatitis Extent and Severity Index; VPS, visual pruritus scale; AKT, protein kinase B; BCL-2, B-cell lymphoma-2; TSLP, thymic stromal lymphopoietin; Tβ-Fb, transforming growth factor-beta-induced fibroblast; connective tissue growth factor (CTGF); SMAD2, suppressed the activation of mothers against decapentaplegic homolog 2; LSF, localized scleroderma-fibroblasts; COL1, collagen type I; COL3, collagen type III; α-SMA, alpha-smooth muscle actin; IGA, Investigator’s Global Assessment.

Hall et al. (30) study showed that intravenously administered ADMSCs at a dose of 1.3 million cells/kg did not have a significant impact on the clinical signs of canine AD or the owner-reported pruritus level. No side effects were spotted during this research. Canines’ skin was assessed for skin lesions: on the collection, administration days from 2 to 3 weeks, 6 to 8 weeks, and from 10 to 12 weeks after ADMSCs implementation. The staging of the severity of skin lesions were evaluated thanks to the Canine Atopic Dermatitis Extent and Severity Index (CADESI-03) and visual pruritus scale (VPS). After the period of twelve weeks the owner of one canine, described a minimal sense of pruritus and minimal appearance of atopic lesions in canine’s skin. However, the study focused on the canine skin, thus findings might not be directly applicable to other species due to variations in skin structure and physiology (30) (**Table 2**).

#### 3.2.2 ADMSCs-derived small extracellular vesicles

Cho et al. (31) showed that canine ADMSC-derived extracellular vesicles (cASC-EVs) have a therapeutic potential for AD. cASC-EVs were effective in alleviating AD-like dermatitis symptoms like skin barrier defects, inflammation and itchiness. Moreover, they observed a reduction in serum IgE levels, which are often elevated in allergic conditions like AD. Additionally, cASC-EVs helped to reduce ear thickness, suggesting a beneficial effect on skin inflammation. Finally, the levels of IL-4 and IFN-γ, two key pro-inflammatory cytokines involved in AD, were significantly decreased following cASC-EV treatment (31) (**Table 2**).

Similarly, Kim et al. (32) presented the therapeutic potential of cASC-EVs for AD. They found a significant reduction in various inflammatory cytokines associated with AD, including IL-4, IL-13, IL-31, RANTES, and TARC. Furthermore, cASC-EVs promoted skin barrier repair by reducing transepidermal water loss, increasing stratum corneum hydration, and upregulating the expression of epidermal differentiation proteins (protein kinase B (AKT) and B-cell lymphoma-2 (BCL-2)). In addition, the study demonstrated that cASC-EVs reduced IL-31/TRPA1-mediated pruritus, a common symptom of AD. Finally, cASC-EVs inhibited the activation of the JAK/STAT signaling pathway, which plays a crucial role in AD inflammation. While mice are a convenient and commonly used model in research, it’s crucial to acknowledge the inherent limitations of extrapolating findings from mice to humans (32) (**Table 2**).

Cho et al. (33) demonstrated that ADMSC-derived exosomes represent a promising cell-free therapeutic option for the treatment of atopic dermatitis. The therapy attenuated atopic dermatitis symptoms by reducing serum IgE levels, eosinophil count, and infiltration of immune cells in skin lesions. ADMSC-exosomes also significantly decreased the *mRNA* expression of multiple pro-inflammatory cytokines (e.g. IL-4, IL-23, IL-31, TNF-α) in AD skin lesions. The study limitation highlights that the variability between different donors hasn’t been fully addressed. This means that the specific characteristics of each donor, like their age, health, and other individual factors, could influence the study’s results, and this potential impact needs further investigation (33) (**Table 2**).

Furthermore, Shin et al. (34) noticed that human ADMSC-exosomes promoted epidermal barrier repair in patients dealing with AD. Subcutaneous injection of ADMSC-exosomes enhanced the formation of lamellar bodies and the lamellar layer in the skin. Moreover, exosomes from human ADMSCs restored the expression of genes involved in the skin barrier, lipid metabolism, cell cycle, and inflammatory response in the diseased area. The cell-free therapy markedly reduced the levels of inflammatory cytokines like IL-4, IL-5, IL-13, TNF-α, IFN-γ, IL-17, and thymic stromal lymphopoietin (TSLP). Finally, ADMSC-exosomes induced the synthesis of ceramides and dihydroceramides, which are essential components of the skin barrier (34) (**Table 2**).

Roh et al. (35) revealed that ADMSC-exosomes can serve as a therapeutic agent for particulate matter (PM)-exacerbated AD. PM exposure led to an upregulation of IL-6, IL-1β, and IL-1α, which are pro-inflammatory cytokines. The levels of IL-10, an anti-inflammatory cytokine, were reduced by PM exposure, similarly the expression of loricrin and filaggrin, key proteins involved in skin barrier function, was decreased. In addition when cells exposed to PM were treated with ADMSC-exosomes, the levels of pro-inflammatory cytokines (IL-6, IL-1β, and IL-1α) were significantly reduced. ADMSC-exosomes helped restore the levels of IL-10, the anti-inflammatory cytokine and the expression of loricrin and filaggrin was upregulated, suggesting a restoration of skin barrier function. Moreover, the expression of FLG was restored, indicating improved skin barrier function. Their results confirmed that PM-induced inflammation and skin barrier damage were alleviated by ADMSC-exosomes in AD-like triple-cell model. The limitation of the research is an *in vitro* used model. *In vitro* studies, while valuable for initial investigation, are conducted in a controlled laboratory environment outside the context of a living organism. Therefore, researchers need to investigate the mechanisms of action of ADMSC-exosomes in AD and their therapeutic potential in animal models and clinical studies (35) (**Table 2**).

Park et al. (36) reported that ADMSCs-derived exosomes, when applied topically with electroporation assistance, may be an effective treatment for dupilumab facial redness. This is based on a case study of two patients with AD who experienced improvement in their erythematous facial lesions after six repeated sessions of this treatment, performed with an interval of 1 week between each session. The small sample size (two patients) significantly weakens the conclusions that can be drawn from the study and limits its impact in the field (36) (**Table 2**).

### 3.3 Systemic sclerosis

Rozier et al. (37) suggests that ADMSC-derived sEVs may have therapeutic potential for fibrotic diseases. They found that ADMSC-derived EVs had anti-fibrotic and pro-remodeling effects on transforming growth factor-beta-induced fibroblasts (Tβ-Fb), which play a crucial role in the development of fibrosis. Additionally, ADMSC-derived EVs were more effective than ADMSCs in improving the myofibroblastic phenotype, shown by the decreased expression of *COL1A1, COL3A1, αSMA*, *IL-6* and the upregulation of *MMP1/TIMP1.* A crucial weakness in the research was the small sample size (2 participants). A study with only two participants lacks sufficient statistical power and generalizability (37) (**Table 2**).

In another study by Kim et al. (38) ADMSC-EVs decreased both the thickness of dermal tissue and the number of (α-SMA) expressing cells, which are markers of fibrosis. Besides, ASC-EVs reduced the mRNA levels of several fibrotic genes, including *Acta2*, *Ccn2*, *Col1a1*, and *Comp*. As well as that, ASC-EVs decreased the expression of α-SMA and connective tissue growth factor (CTGF), two key players in the TGF-β pathway, and suppressed the activation of mothers against decapentaplegic homolog 2 (SMAD2), a downstream effector of TGF-β signaling. Lastly, ASC-EVs induced selective death in dermal fibroblasts stimulated with TGF-β1. The major limitations of the study were animal model used and small sample size (38) (**Table 2**).

### 3.4 Scleroderma

Wang et al. (39) showed that ADMSC-exosomes can prevent the bioactivity of localized scleroderma-fibroblasts (LSF). Namely, ADMSC-exosomes reduced collagen deposition and myofibroblast trans-differentiation in LSF. The downregulation of COL1 (type I collagen), COL3 (type III collagen), α-SMA (alpha-smooth muscle actin), and p-Smad2/3 by ADMSC-Exo suggests that they may interfere with the TGF-β signaling pathway, which is known to play a crucial role in fibrosis. TGF-βR1 (transforming growth factor-beta receptor 1) knockdown and let-7a-5p mimic (a miRNA that targets TGF-βR1) in LSFs also led to a reduction in COL1, COL3, α-SMA, and p-Smad2/3 (phosphorylated Smad2/3) expression, further supporting the involvement of the TGF-β pathway. The limitation of this study was based on localized scleroderma-derived fibroblasts. Results from localized scleroderma fibroblasts might not reflect the behavior of fibroblasts in systemic sclerosis (39) (**Table 2**).

### 3.5 Acne vulgaris

Kwon et al. (40) reported that the ADMSC-exosome-treated sides showed a greater reduction in acne scar severity compared to control sides. By increasing collagen synthesis, ADMSC-exosomes can help to improve skin thickness, texture, and firmness. Moreover, ADMSC-exosomes can stimulate the movement and growth of skin cells, which is essential for wound healing and tissue regeneration. In turn, by reducing the levels of inflammatory cytokines, ADMSC-exosomes can help to reduce inflammation and promote healing. The ADMSC-exosome-treated facial side demonstrated superior improvement compared to the control side after 3 sessions of each treatment, as assessed by the Investigator’s Global Assessment (IGA) score (p = 0.02). At the final follow-up visit, all of the following parameters were significantly decreased from baseline on the ADMSC-exosome side: atrophic scar volume, mean pore volume, skin surface roughness. Various treatment-related side effects, including post-treatment pain, erythema, edema, and dryness, were experienced on both ADMSC-exosome and control sides, but they were nearly resolved within 5 days. The study’s limitation is the small sample size of 25 patients, with a disproportionate representation of males (18) compared to females (7). This imbalance could affect the generalizability of the findings (40) (**Table 2**).

## 4 Conclusions

ADMSCs and ADMSC-derived small extracellular vesicles are promising candidates for the treatment of immune-mediated inflammatory skin diseases. Research suggests that ADMSCs can migrate to skin lesions, limit autoimmunity, and suppress inflammation, potentially leading to improved outcomes for psoriasis and AD. Although preclinical and clinical studies have shown that ADMSC transplantation is safe and tolerable, large clinical trials are needed to investigate the long-term safety and efficacy of this approach. Relatively few studies have been published to date, therefore further studies are needed to address some of the issues, including standardization of cell processing and culture protocols, ideal route of transplantation, dosage and timing of ADMSC administration. Accumulating evidence suggests that sEVs exhibit immunoregulatory functions similar to those of parental MSCs, which can be administered systemically with minimal toxicity. Considering the available studies, ADMSC-sEVs may be a new feasible option for the treatment of common skin diseases in addition to cell-based therapy.

## Data Availability

The original contributions presented in the study are included in the article/supplementary material. Further inquiries can be directed to the corresponding author.
